# The association between use of online social networks sites and perceived social isolation among individuals in the second half of life: results based on a nationally representative sample in Germany

**DOI:** 10.1186/s12889-018-6369-6

**Published:** 2019-01-09

**Authors:** André Hajek, Hans-Helmut König

**Affiliations:** Department of Health Economics and Health Services Research, Hamburg Center for Health Economics, University Medical Center Hamburg-Eppendorf, Martinistr. 52, 20246 Hamburg, Germany

**Keywords:** Social isolation, Social media use, Social exclusion, Germany

## Abstract

**Background:**

To date, little is known about the association between the use of online social network sites and social isolation among individuals in the second half of life. Therefore, the aim of the present study was to examine this association among older adults.

**Methods:**

Cross-sectional data was drawn from a nationally representative sample of non-institutionalized individuals aged 40 and above (*n* = 7837) in Germany (German Ageing Survey). Online social network use was assessed using the frequency of social network use (e.g., Facebook) in the preceding 12 months (daily; several times a week; once a week; 1–3 times a month; less often; never). Perceived social isolation was measured using an established scale created by Bude and Lantermann.

**Results:**

Adjusting for covariates, linear regressions revealed that daily online social network users reported *lower* social isolation scores compared with those with less frequent or no social media use.

**Conclusions:**

Data suggest that daily users of online social networks aged 40 and over tend to feel *less* socially isolated than less frequent users or non-users. Future research should concentrate on identifying the direction of this association. Moreover, the reasons underlying this finding should be examined.

**Electronic supplementary material:**

The online version of this article (10.1186/s12889-018-6369-6) contains supplementary material, which is available to authorized users.

## Background

Since the early 2000s, online social network sites (e.g., Facebook) have become increasingly popular throughout the world. This is particularly true among young adolescents and young adults. However, use of social network sites is also increasing among middle aged and older adults. As a number of those aged 40 years and older are already familiar with the internet and online social networks, it is likely that the proportion of individuals in old age using social networks will increase in the coming decades.

It has recently been demonstrated in a study of adults in the U. S. aged 19 to 32 years, that users of social media (e.g., Facebook) report higher perceived social isolation (used interchangeably: social exclusion) [1]. Social isolation is a feeling that one does not belong to the society. A positive association between the use of social network sites and loneliness (the perceived discrepancy between actual and desired social relationships) has also been shown among younger adults in different countries [2, 3]. It is worth noting that while loneliness is related to social isolation, these are two distinct constructs [4] and they differ in their correlates [5].

To date, little is known about the association between the use of online social networks and social isolation among individuals in the second half of life. Previous studies focusing on older adults were interested in the relationship between social network sites and *loneliness*. For example, a recent study conducted by Aarts and colleagues on community-dwelling older adults ≥60 years in the Netherlands (*n* = 626) showed that the use of online social network sites was neither related to loneliness in general, nor was it related to emotional and social loneliness [6]. In contrast, Leist concluded in a review that social media can reduce loneliness among older adults [7]. She stated that online communities are “places where people can get together and engage in social contact, e.g. overcome loneliness at nighttime” [7].

In order to close the gap in knowledge, the purpose of this study was to examine the link between the use of online social networks such as Facebook and social isolation among individuals aged 40 and over in Germany, using a nationally representative sample. Understanding this association is important due to the well-established association between social isolation, and morbidity as well as mortality [8].

Frequent users of social network sites may replace real life social interactions with these sites. Moreover, the frequent use of these sites may lead users to perceive that others have more, or better quality, social relationships than themselves, due to the unrealistic portrayals of reality on social network sites [1]. On the other hand, individuals who perceive themselves as socially isolated may feel less isolated when using social network sites, as these sites may facilitate relationship building by enhancing social ties [9, 10].

## Methods

### Sample

Data was drawn from the German Ageing Survey (German language: “Deutscher Alterssurvey”, DEAS), a longitudinal cohort-based survey of non-institutionalized individuals ≥40 years in Germany. Data collection took place in 1996 (first wave), 2002 (second wave), 2008 (third wave), 2011 (fourth wave), and 2014 (fifth wave). Both, cross-sectional and panel samples were included in the follow-up waves (with the exception of 2011, which was a pure panel survey). The response rate in 2014 was approximately 61% and 25% for the panel and the cross-sectional sample, respectively. This response rate is comparable to other large survey studies conducted in Germany [11], and similarly reflects the trend of decreasing rates in participation in surveys in Germany. It is worth noting that intense efforts were made to mitigate this trend in the DEAS study.

In this study, only data from the fifth wave was used, as both variables of interest (use of social networks in the internet and social isolation) were *not* assessed in previous waves. Further details with regard to the DEAS study can be found elsewhere [12].

### Dependent variables

A scale created by Bude and Lantermann [13] was used to assess social isolation. This scale consists of four items, namely “I feel excluded from society”, “I am worried to be left behind”, “I feel that I am left out” and “I feel like I do not really belong to society”. Respondents are asked to rate the strength of their agreement with the item from 1 to 4 (1 = “strongly agree” to 4 = “strongly disagree”). All items were recoded. The score comprises the mean of at least 2 of the items, with higher values corresponding to higher social isolation. Consequently, it was considered as continuous variable. This is in line with previous research [14]. In this study, Cronbach’s alpha was .88.

Associations between social isolation and low education, as well as poverty, were demonstrated using this social isolation scale [15]. Moreover, an association between social isolation and use of preventive cancer screenings was identified using this social isolation scale [16].

### Independent variables

Social network use was measured using the frequency of social network use (for example, popular social networks among older adults in Germany such as Facebook, StayFriends or feierabend.de) in the past 12 months (‘daily‘; ‘several times a week‘; ‘once a week‘; ‘1–3 times a month‘; ‘less often‘; ‘never‘).

Age, marital status (married, living together with spouse; other (widowed; divorced; single; married, living separated from spouse)), employment status (employed; retired; other (not employed)), equivalent monthly net income (OECD scale) were adjusted for in the regression analysis. Moreover, lifestyle factors were adjusted for, including the frequency of alcohol consumption, physical activity (in both cases: ‘never’, ‘rarer than once a month’, ‘one to three times a month’, ‘once a week’, ‘several times a week’, and ‘daily’) and smoking habits (non-smoker; former smoker; casual smoker; daily smoker). Self-rated health (ranging from 1 = very good to 5 = very bad) and the number of physical illnesses (hearing problems, ear problems; vision impairment, eye problems; bladder problems; liver or kidney problems; gall bladder; diabetes; cancer; stomach and intestinal problems; respiratory problems, asthma, shortness of breath; joint, bone, spinal or back problems; bad circulation; cardiac and circulatory disorders) were also included as potential confounders in the regression analysis.

In a sensitivity analysis, the robustness of the results was tested by adding depression (Center for Epidemiological Studies Depression Scale, CES-D ≥ 18 [17]) and physical functioning (subscale ‘physical functioning’ of the SF-36 which ranges from 0 (worst) to 100 (best)) to our main model [18]. They were only used in the sensitivity analysis as the direction of the relationship between these variables and social isolation is both unclear and debated (reverse causality).

The main independent variable was replaced with another (broader) explanatory variable in a further analysis. This was carried out to determine whether the association between the use of social network sites and social isolation depends on the measure used to quantify the independent variable. Individuals with access to the internet were asked how often they use the internet for “contact with friends and relatives (e.g. e-mail, Facebook, chat, video calls)” (‘daily‘; ‘several times a week‘; ‘once a week‘; ‘1–3 times a month‘; ‘less often‘; ‘never‘).

Further tests were carried out to determine whether the association between the use of social network sites and social isolation was moderated by educational level.

### Statistical analysis

First, sample characteristics were calculated (stratified by the use of social network sites). Second, multiple linear regressions were performed to examine the association between the use of social network sites and social isolation. Statistical significance was set set at *p* < 0.05. Stata 15.0 (Stata Corp, College Station, TX, USA) was used to conduct statistical analysis. Regression analysis were run for the total sample and stratified by sex.

## Results

### Description of the sample

Sample characteristics, stratified by the use of social network sites, can be found in Table 1 (row percentages included). While daily users were on average 61.0 (±10.3) years old, non-users were 72.0 (±9.4) years old. Less than one out of two of daily users were retired, however approximately four out of five among the non-users were retired. In addition, daily users reported on average 2.3 (±1.7) physical illnesses, whereas non-users reported on average 3.2 (±2.0) physical illnesses. The mean physical functioning among daily users was 86.8 (±18.9), whereas mean physical functioning was 72.1 (±26.8) among non-users. Moreover, whilst 4.6% (139 out of 3002) of the daily users had depression, 8.0% of the non-users (189 out of 2363) had depression. The average social isolation scores among daily users and non-users were 1.5 (±0.5) and 1.7 (±0.7) respectively.

**Table 1:** Sample characteristics stratified by the use of social network sites (German Ageing Survey, fifth wave, *n* = 7837)

	Daily (*n* = 3002; 38.3%)	Several times a week (*n* = 1362; 17.4%)	Once a week (*n* = 422; 5.4%)	1 to 3 times a month (*n* = 180; 2.3%)	Less often (*n* = 508; 6.5%)	Never (*n* = 2363; 30.1%)	*p*-Value
	N (%)/Mean (SD)	N (%)/Mean (SD)	N (%)/Mean (SD)	N (%)/Mean (SD)	N (%)/Mean (SD)	N (%)/Mean (SD)	
Social isolation (from 1 (lowest social isolation) to 4 (highest social isolation))	1.5 (0.5)	1.6 (0.6)	1.6 (0.6)	1.6 (0.6)	1.7 (0.6)	1.7 (0.7)	<.001
Gender: Female	1263 (31.6%)	755 (18.9%)	232 (5.8%)	107 (2.7%)	295 (7.4%)	1344 (33.6%)	<.001
Age in years	61.0 (10.3)	61.3 (10.5)	61.4 (10.3)	61.8 (10.4)	61.7 (9.8)	72.0 (9.4)	<.001
Marital status: - married and living together with spouse	2234 (40.8%)	965 (17.6%)	308 (5.6%)	118 (2.2%)	386 (7.1%)	1464 (26.7%)	<.001
Employment status: - employed	1432 (50.0%)	646 (22.5%)	200 (7.0%)	85 (3.0%)	238 (8.3%)	265 (9.2%)	<.001
- retired	1305 (30.6%)	595 (14.0%)	195 (4.6%)	73 (1.7%)	215 (5.0%)	1879 (44.1%)	
- other: not employed	265 (37.6%)	120 (17.0%)	27 (3.8%)	22 (3.1%)	54 (7.7%)	217 (30.8%)	
Monthly net equivalent income in Euro	2300.5 (1696.8)	2026.0 (1208.8)	1866.9 (957.6)	1738.0 (955.3)	1822.6 (934.7)	1496.4 (1017.8)	<.001
Smoking status: - daily	413 (38.4%)	199 (18.5%)	58 (5.4%)	40 (3.7%)	90 (8.4%)	276 (25.6%)	<.001
- Yes, sometimes	140 (45.3%)	57 (18.4%)	20 (6.5%)	8 (2.6%)	25 (8.1%)	59 (19.1%)	
- Not anymore	1238 (43.0%)	482 (16.7%)	146 (5.1%)	59 (2.1%)	172 (6.0%)	781 (27.1%)	
- Never been smoker	1189 (34.0%)	615 (17.6%)	196 (5.6%)	71 (2.0%)	219 (6.2%)	1210 (34.6%)	
Consumption of alcohol: - daily	414 (44.8%)	146 (15.8%)	46 (5.0%)	13 (1.4%)	56 (6.0%)	250 (27.0%)	<.001
- several times a week	907 (48.2%)	348 (18.5%)	91 (4.8%)	41 (2.2%)	103 (5.5%)	391 (20.8%)	
- once a week	480 (38.6%)	267 (21.5%)	91 (7.3%)	36 (2.9%)	76 (6.1%)	294 (23.6%)	
- one to three times a month	338 (36.1%)	196 (20.9%)	65 (6.9%)	19 (2.0%)	73 (7.8%)	247 (26.3%)	
- less frequently	555 (29.8%)	291 (15.6%)	98 (5.3%)	51 (2.7%)	138 (7.4%)	729 (39.2%)	
- never	268 (30.7%)	101 (11.6%)	29 (3.3%)	17 (1.9%)	53 (6.1%)	406 (46.4%)	
Physical activity: - daily	284 (42.9%)	95 (14.3%)	36 (5.4%)	11 (1.7%)	42 (6.3%)	195 (29.4%)	<.001
- several times a week	988 (46.3%)	488 (22.9%)	125 (5.8%)	36 (1.7%)	122 (5.7%)	376 (17.6%)	
- once a week	570 (40.0%)	256 (17.9%)	83 (5.8%)	38 (2.7%)	126 (8.8%)	354 (24.8%)	
- one to three times a month	242 (40.9%)	115 (19.4%)	33 (5.6%)	20 (3.4%)	46 (7.7%)	136 (23.0%)	
- less frequently	380 (41.4%)	159 (17.3%)	63 (6.9%)	21 (2.3%)	74 (8.0%)	221 (24.1%)	
- never	538 (25.6%)	249 (11.9%)	82 (3.9%)	53 (2.5%)	98 (4.7%)	1081 (51.4%)	
Self-rated health (from 1 = “very good” to 5 = “very bad”)	2.4 (0.8)	2.4 (0.8)	2.5 (0.9)	2.4 (0.9)	2.5 (0.8)	2.7 (0.8)	<.001
Number of physical illnesses (from 0 to 11)	2.3 (1.7)	2.4 (1.8)	2.3 (1.7)	2.4 (1.8)	2.5 (1.8)	3.2 (2.0)	<.001
Depression (CES-D ≥ 18)	139 (28.4%)	86 (17.6%)	30 (6.1%)	12 (2.5%)	33 (6.7%)	189 (38.7%)	<.001
Physical functioning (from 0 = lowest score to 100 = best score)	86.8 (18.9)	85.4 (19.9)	84.7 (20.9)	84.9 (19.0)	82.5 (22.0)	72.1 (26.8)	<.001

Comments: Row percentages were presented in this table. Social isolation was quantified using a scale developed by Bude and Lantermann [13]; Depression was quantified using the CES-D [17]. Physical functioning was quantified using the subscale ‘physical functioning’ of the SF-36 [18]

### Regression analysis

In an adjusted analysis, the association between the use of online social network sites and social isolation was investigated (first column: total sample; second column: men; third column: women; Table 2).

**Table 2:** Determinants of social isolation. Results of multiple linear regression analysis (German Ageing Survey, fifth wave)

Independent variables	(1)	(2)	(3)
	Total sample	Men	Women
Use of social network sites: - Several times a week (Ref.: daily)	0.05**	0.06*	0.04
	(0.02)	(0.03)	(0.03)
- Once a week	0.07*	0.12**	0.04
	(0.03)	(0.04)	(0.04)
- 1 to 3 times a month	0.05	0.10	0.02
	(0.05)	(0.08)	(0.06)
- Less often	0.10***	0.09*	0.09*
	(0.03)	(0.04)	(0.04)
- Never	0.10***	0.12***	0.07*
	(0.02)	(0.03)	(0.03)
Age	−0.01***	−0.01***	− 0.01***
	(0.00)	(0.00)	(0.00)
Marital status: Other (divorced, widowed, single, married, living separated from spouse) (Ref.: married and living together with spouse)	0.08***	0.11***	0.05*
	(0.02)	(0.02)	(0.02)
Employment status: - Retired (Ref.: employed)	0.08***	0.10**	0.06+
	(0.02)	(0.03)	(0.03)
- Other: not employed	0.17***	0.19***	0.15***
	(0.03)	(0.05)	(0.04)
Monthly net equivalent income in 1000 Euro	−0.04***	−0.04***	− 0.05***
	(0.01)	(0.01)	(0.01)
Smoking status: - Yes, sometimes (Ref.: Daily)	−0.07*	− 0.02	− 0.13*
	(0.03)	(0.05)	(0.05)
- Not anymore	−0.01	−0.02	0.01
	(0.02)	(0.03)	(0.03)
- Never been smoker	−0.03	−0.04	0.00
	(0.02)	(0.03)	(0.03)
Consumption of alcohol: - Several times a week (Ref.: Daily)	0.00	0.01	−0.03
	(0.02)	(0.03)	(0.04)
- Once a week	0.01	0.06*	−0.09*
	(0.02)	(0.03)	(0.04)
- 1 to 3 times a month	0.04+	0.08*	−0.03
	(0.03)	(0.04)	(0.04)
- Less often	0.06*	0.09*	0.00
	(0.02)	(0.03)	(0.04)
- Never	0.11***	0.15***	0.04
	(0.03)	(0.04)	(0.05)
Physical activity: - Several times a week (Ref.: Daily)	−0.03	−0.04	−0.03
	(0.02)	(0.04)	(0.03)
- Once a week	0.00	−0.02	0.02
	(0.03)	(0.04)	(0.04)
- 1 to 3 times a month	0.03	−0.01	0.07
	(0.03)	(0.04)	(0.05)
- Less often	−0.01	−0.04	0.02
	(0.03)	(0.04)	(0.04)
- Never	0.02	0.00	0.03
	(0.03)	(0.04)	(0.04)
Self-rated health (from 1 = “very good” to 5 = “very bad”)	0.10***	0.11***	0.09***
	(0.01)	(0.01)	(0.01)
Number of physical illnesses (from 0 to 11)	0.05***	0.04***	0.05***
	(0.00)	(0.01)	(0.01)
Constant	1.60***	1.52***	1.71***
	(0.07)	(0.10)	(0.10)
Observations	7141	3549	3592
R^2^	0.12	0.14	0.10

Comments: Beta-Coefficients are reported; robust standard errors in parentheses. *** *p* < 0.001, ** *p* < 0.01, * p < 0.05, + *p* < 0.10. Social isolation was quantified using a scale developed by Bude and Lantermann [13]

Compared to daily users, less frequent users (e.g., several times a week (total sample): β = .05, *p* < .01) and non-users reported statistically significant higher social isolation scores in the total sample and in men (except for the group “1 to 3 times a month”). In women, the “less often” (β = .09, *p* < .05) and “never” (β = .07, *p* < .05) groups reported statistically significant higher social isolation scores compared to daily users. However, it is worth noting that gender differences (sex x use of social network sites) were not significant, with the exception of the interaction term “never” x sex (β = −.08, *p* = .02).

As for the covariates (first column), social isolation was consistently positively associated with not being married, living together with spouse, being unemployed, lower income, lower age, poorer self-rated health as well as more physical illnesses in the total sample and in both sexes. Please see Table 2 for further details.

In a sensitivity analysis (see Additional file 1), the main model was extended by adding depression and physical functioning as covariates. In terms of effect size and significance, the association between the use of social network sites and social isolation remained almost the same in the total sample and in both sexes.

In a second sensitivity analysis, it was tested whether educational level moderates the association of interest (by including interaction terms educational level x use of social network sites). None of the interaction terms achieved statistical significance (see Additional file 2).

A third sensitivity analysis was conducted, where the main independent variable was replaced with the variable ‘frequency of internet use for contact with friends and relatives (e.g. e-mail, Facebook, chat, video calling)’. The results were similar to those obtained in the prior regression analyses. Compared to daily users, less frequent users and non-users reported statistically significant higher social isolation scores (see Additional file 3).

## Discussion

Drawing on a nationally representative sample of non-institutionalized adults ≥40 years, the objective of this study was to examine the association between the use of online social network sites and social isolation. Interestingly, non-users of social networks site were markedly older, more functionally impaired and had a higher number of physical illnesses compared to (daily) users in our study. Multiple linear regressions revealed that compared to daily users, less frequent users and non-users reported statistically significant higher social isolation scores.

To date, there is equivocal evidence concerning the association between the use of social networks sites and social isolation. The variance in results outlined in the introduction section can mainly be explained by differences in measuring the independent variable of interest (use of online social network sites), differences in the outcome measure (loneliness vs. social isolation) and large differences in the sample composition (e.g., student samples vs. community-dwelling older adults). Moreover, we assume that age is associated with the motivation to use online social network sites, which may influence the strength of association between their use and social isolation.

How can our findings be explained? Intuitively, one might assume that time spent on social networks replaces real life social experiences, which are associated with actual decreases in social isolation [1]. Moreover, use of social network sites may facilitate comparisons of oneself to idealized portrayals of others, or may highlight instances where one is not invited to an event and/or social gathering. Such instances may elicit feelings of envy and may result in social isolation [1]. Realistic evaluation of images and events viewed via such sites may lead to the opposite response by the user. That is, the user may conclude that he or she is better off socially than others are, leading to decreased social isolation. This possibility could explain our results. Another way to explain our findings might be that daily users in the second half of life might compare with less frequent or non-users. Daily users might also conclude that they are better off than the latter groups because they are still able to participate in social network sites which in turn might reflect a better perceived health status compared to the latter groups [19, 20]. This might result in decreased social isolation. It appears plausible that daily users have a better health status compared to non-users. In Table 1, it was, for example, shown that daily users reported better health (in terms of self-rated health, number of physical illnesses, depression or physical functioning).

This study has several strengths. To our knowledge, this is the first study investigating the relation between the use of social network sites and social isolation based on a nationally representative sample of community-dwelling individuals in the second half of life in Germany. Social isolation was quantified using an established scale developed by Bude and Lantermann. It was adjusted for various potential confounders, including socioeconomic, lifestyle and health-related factors. The frequency of social network use in the past 12 months was used to quantify the social network use. The robustness of our findings was checked by replacing this variable with a broader variable covering the frequency of internet use for contact with friends and relatives in general. However, further research that assesses the time spent using social network sites is required. As this is a cross-sectional study, changes within individuals over time could not be examined. Further research is needed to clarify the direction of this relationship. In addition, a reasonably small sample selection bias has been reported in the DEAS study [12].

## Conclusion

Unexpectedly, data suggest that daily users of online social network sites aged 40 and over tend to feel *less* socially isolated than less frequent users or non-users. Future research should concentrate on identifying the direction of this association. Moreover, the reasons underlying these findings should be examined. The association could differ by personality type, cultural settings, and/or geographical location. Furthermore, the type of social media use (e.g., distinguishing between passive consumers and active users) [9] should be investigated in depth.

## Additional files

Additional file 1: Determinants of social isolation. Results of multiple linear regression analysis (German Ageing Survey, fifth wave) (with additional control variables). (DOCX 16 kb)

Additional file 2: Determinants of social isolation. Results of multiple linear regression analysis (with interaction terms: use of social network sites x education). (DOCX 16 kb)

Additional file 3: Determinants of social isolation. Results of multiple linear regression analysis (German Ageing Survey, fifth wave) (with another main independent variable). (DOCX 16 kb)

## Abbreviations

CES-D: Center for Epidemiological Studies Depression Scale
DEAS: German Ageing Survey
OECD: Organization for Economic Co-Operation and Development
