# Supplementary material for: The association between use of online social networks sites and perceived social isolation among individuals in the second half of life: results based on a nationally representative sample in Germany
Source: BMC Public Health. 2019 Jan 9;19:40. doi: 10.1186/s12889-018-6369-6 (PMC6325850; doi:10.1186/s12889-018-6369-6)
Supplement: Supplementary file 2 — Determinants of social isolation. Results of multiple linear regression analysis (with interaction terms: use of social network sites x education). (DOCX 16 kb) [file 12889_2018_6369_MOESM2_ESM.docx]

Additional file 2. Determinants of social isolation. Results of multiple linear regression analysis (German Ageing Survey, fifth wave) (with interaction terms: use of social network sites x education)

|  | (1) | (2) | (3) |
| --- | --- | --- | --- |
| Independent variables | Total sample | Men | Women |
|  |  |  |  |
| Use of social network sites: - Several times a week (Ref.: daily) | 0.01 | 0.02 | -0.02 |
|  | (0.03) | (0.05) | (0.04) |
| - Once a week | 0.05 | 0.14+ | -0.01 |
|  | (0.05) | (0.08) | (0.06) |
| - 1 to 3 times a month | -0.00 | 0.07 | -0.06 |
|  | (0.08) | (0.14) | (0.10) |
| - Less often | 0.04 | 0.07 | 0.01 |
|  | (0.04) | (0.07) | (0.06) |
| - Never | 0.08** | 0.10* | 0.06 |
|  | (0.03) | (0.04) | (0.04) |
| Education: - Low (Ref.: middle) | 0.09 | -0.02 | 0.15 |
|  | (0.12) | (0.17) | (0.16) |
| Education: - High | -0.02 | 0.00 | -0.05 |
|  | (0.03) | (0.04) | (0.04) |
| Interaction term: Use of social network sites: - Several times a week (Ref.: daily) x low education (Ref.: middle) | 0.19 | 0.34+ | 0.12 |
|  | (0.22) | (0.19) | (0.28) |
| Interaction term: Use of social network sites: - Several times a week (Ref.: daily) x high education (Ref.: middle) | 0.05 | 0.02 | 0.10 |
|  | (0.05) | (0.07) | (0.08) |
| Interaction term: Use of social network sites: - Once a week (Ref.: daily) x low education (Ref.: middle) | 0.15 | 0.36 | 0.03 |
|  | (0.25) | (0.36) | (0.32) |
| Interaction term: Use of social network sites: - Once a week (Ref.: daily) x high education (Ref.: middle) | 0.05 | -0.01 | 0.09 |
|  | (0.08) | (0.11) | (0.11) |
| Interaction term: Use of social network sites: - 1 to 3 times a month (Ref.: daily) x low education (Ref.: middle) | 0.26+ | 0.31 | 0.21 |
|  | (0.15) | (0.25) | (0.17) |
| Interaction term: Use of social network sites: - 1 to 3 times a month (Ref.: daily) x high education (Ref.: middle) | - | - | - |
|  | - | - | - |
| Interaction term: Use of social network sites: - Less often (Ref.: daily) x low education (Ref.: middle) | 0.18 | 0.32 | 0.06 |
|  | (0.17) | (0.25) | (0.22) |
| Interaction term: Use of social network sites: - Less often (Ref.: daily) x high education (Ref.: middle) | -0.00 | -0.08 | 0.10 |
|  | (0.09) | (0.12) | (0.12) |
| Interaction term: Use of social network sites: - Never (Ref.: daily) x low education (Ref.: middle) | -0.09 | 0.08 | -0.15 |
|  | (0.13) | (0.21) | (0.18) |
| Interaction term: Use of social network sites: - Never (Ref.: daily) x high education (Ref.: middle) | -0.03 | -0.08 | 0.04 |
|  | (0.06) | (0.08) | (0.09) |
| Age | -0.01*** | -0.00* | -0.01*** |
|  | (0.00) | (0.00) | (0.00) |
| Marital status: Other (divorced, widowed, single, married, living separated from spouse) (Ref.: married and living together with spouse) | 0.08*** | 0.13*** | 0.05+ |
|  | (0.02) | (0.03) | (0.03) |
| Employment status: - Retired (Ref.: employed) | 0.07* | 0.07 | 0.07 |
|  | (0.03) | (0.05) | (0.05) |
| - Other: not employed | 0.15*** | 0.13+ | 0.14** |
|  | (0.04) | (0.07) | (0.05) |
| Monthly net equivalent income in 1,000 Euro | -0.04*** | -0.03*** | -0.05*** |
|  | (0.01) | (0.01) | (0.01) |
| Smoking status: - Yes, sometimes (Ref.: Daily) | -0.06 | -0.07 | -0.06 |
|  | (0.04) | (0.06) | (0.07) |
| - Not anymore | 0.00 | -0.06 | 0.07 |
|  | (0.03) | (0.04) | (0.04) |
| - Never been smoker | -0.01 | -0.05 | 0.04 |
|  | (0.03) | (0.04) | (0.04) |
| Consumption of alcohol: - Several times a week (Ref.: Daily) | 0.00 | 0.03 | -0.07 |
|  | (0.03) | (0.04) | (0.06) |
| - Once a week | 0.02 | 0.08+ | -0.10+ |
|  | (0.03) | (0.04) | (0.06) |
| - 1 to 3 times a month | 0.05 | 0.09+ | -0.04 |
|  | (0.04) | (0.05) | (0.06) |
| - Less often | 0.07* | 0.10* | -0.00 |
|  | (0.03) | (0.05) | (0.06) |
| - Never | 0.15*** | 0.20** | 0.07 |
|  | (0.04) | (0.06) | (0.07) |
| Physical activity: - Several times a week (Ref.: Daily) | -0.03 | -0.03 | -0.02 |
|  | (0.03) | (0.05) | (0.05) |
| - Once a week | 0.01 | -0.01 | 0.03 |
|  | (0.04) | (0.05) | (0.05) |
| - 1 to 3 times a month | 0.04 | -0.01 | 0.09 |
|  | (0.04) | (0.06) | (0.07) |
| - Less often | -0.00 | -0.03 | 0.02 |
|  | (0.04) | (0.05) | (0.06) |
| - Never | 0.02 | 0.01 | 0.03 |
|  | (0.04) | (0.05) | (0.05) |
| Self-rated health (from 1 = “very good” to 5 = “very bad”) | 0.10*** | 0.11*** | 0.09*** |
|  | (0.01) | (0.02) | (0.02) |
| Number of physical illnesses (from 0 to 11) | 0.05*** | 0.05*** | 0.05*** |
|  | (0.01) | (0.01) | (0.01) |
| Constant | 1.55*** | 1.41*** | 1.72*** |
|  | (0.10) | (0.13) | (0.14) |
|  |  |  |  |
| Observations | 3,830 | 1,921 | 1,909 |
| R² | 0.13 | 0.15 | 0.12 |

Comments: Beta-Coefficients are reported; robust standard errors in parentheses. *** p<0.001, ** p<0.01, * p<0.05, + p<0.10. Social isolation was quantified using a scale developed by Bude and Lantermann [13]. The interaction term for “Use of social network sites: - 1 to 3 times a month (Ref.: daily)” and “high education (Ref.: middle)” identifies no observations in the sample.
