# Supplementary material for: The association between use of online social networks sites and perceived social isolation among individuals in the second half of life: results based on a nationally representative sample in Germany
Source: BMC Public Health. 2019 Jan 9;19:40. doi: 10.1186/s12889-018-6369-6 (PMC6325850; doi:10.1186/s12889-018-6369-6)
Supplement: Supplementary file 3 — Determinants of social isolation. Results of multiple linear regression analysis (German Ageing Survey, fifth wave) (with another main independent variable). (DOCX 16 kb) [file 12889_2018_6369_MOESM3_ESM.docx]

Additional file 3. Determinants of social isolation. Results of multiple linear regression analysis (German Ageing Survey, fifth wave) (with another main independent variable)

|  | (1) | (2) | (3) |
| --- | --- | --- | --- |
| Independent variables | Total sample | Men | Women |
|  |  |  |  |
| Use of the internet for contact with friends and relatives: - Several times a week (Ref.: daily) | 0.07** | 0.07* | 0.07* |
|  | (0.02) | (0.03) | (0.03) |
| - Once a week | 0.07** | 0.08* | 0.06 |
|  | (0.03) | (0.04) | (0.04) |
| - 1 to 3 times a month | 0.09** | 0.08* | 0.09* |
|  | (0.03) | (0.04) | (0.04) |
| - Less often | 0.11*** | 0.12*** | 0.11** |
|  | (0.02) | (0.03) | (0.04) |
| - Never | 0.08** | 0.08* | 0.07+ |
|  | (0.03) | (0.04) | (0.04) |
| Age | -0.01*** | -0.00* | -0.01*** |
|  | (0.00) | (0.00) | (0.00) |
| Marital status: Other (divorced, widowed, single, married, living separated from spouse) (Ref.: married and living together with spouse) | 0.09*** | 0.14*** | 0.06* |
|  | (0.02) | (0.03) | (0.02) |
| Employment status: - Retired (Ref.: employed) | 0.04 | 0.04 | 0.04 |
|  | (0.03) | (0.04) | (0.04) |
| - Other: not employed | 0.16*** | 0.15* | 0.14*** |
|  | (0.03) | (0.06) | (0.04) |
| Monthly net equivalent income in Euro | -0.00*** | -0.00*** | -0.00*** |
|  | (0.00) | (0.00) | (0.00) |
| Smoking status: - Yes, sometimes (Ref.: Daily) | -0.07+ | 0.00 | -0.15** |
|  | (0.04) | (0.05) | (0.06) |
| - Not anymore | -0.02 | -0.02 | -0.01 |
|  | (0.02) | (0.03) | (0.04) |
| - Never been smoker | -0.03 | -0.03 | -0.02 |
|  | (0.02) | (0.03) | (0.04) |
| Consumption of alcohol: - Several times a week (Ref.: Daily) | -0.00 | 0.01 | -0.04 |
|  | (0.02) | (0.03) | (0.05) |
| - Once a week | -0.00 | 0.05 | -0.10* |
|  | (0.03) | (0.04) | (0.05) |
| - 1 to 3 times a month | 0.04 | 0.07+ | -0.04 |
|  | (0.03) | (0.04) | (0.05) |
| - Less often | 0.07* | 0.10** | -0.01 |
|  | (0.03) | (0.04) | (0.05) |
| - Never | 0.08* | 0.12* | 0.00 |
|  | (0.04) | (0.05) | (0.06) |
| Physical activity: - Several times a week (Ref.: Daily) | -0.05 | -0.03 | -0.06 |
|  | (0.03) | (0.04) | (0.04) |
| - Once a week | 0.00 | -0.02 | 0.02 |
|  | (0.03) | (0.04) | (0.04) |
| - 1 to 3 times a month | 0.03 | 0.01 | 0.07 |
|  | (0.04) | (0.05) | (0.05) |
| - Less often | -0.03 | -0.05 | -0.02 |
|  | (0.03) | (0.04) | (0.05) |
| - Never | 0.03 | 0.03 | 0.03 |
|  | (0.03) | (0.04) | (0.05) |
| Self-rated health (from 1 = “very good” to 5 = “very bad”) | 0.10*** | 0.10*** | 0.09*** |
|  | (0.01) | (0.02) | (0.02) |
| Number of physical illnesses (from 0 to 11) | 0.05*** | 0.04*** | 0.05*** |
|  | (0.01) | (0.01) | (0.01) |
| Constant | 1.49*** | 1.34*** | 1.69*** |
|  | (0.08) | (0.11) | (0.12) |
|  |  |  |  |
| Observations | 5,054 | 2,592 | 2,462 |
| R² | 0.12 | 0.13 | 0.11 |

Comments: The main independent variable (“Use of social network sites”) was replaced by the variable “use of the internet for contact with friends and relatives”. Beta-Coefficients are reported; robust standard errors in parentheses. *** p<0.001, ** p<0.01, * p<0.05, + p<0.10. Social isolation was quantified using a scale developed by Bude and Lantermann (13); Depression was quantified using the CES-D (8). Physical functioning was quantified using the subscale ‘physical functioning’ of the SF-36 (9).
